# Draft Genome Sequence of *Bacillus* sp. FMQ74, a Dairy-Contaminating Isolate from Raw Milk

**DOI:** 10.1128/genomeA.01512-16

**Published:** 2017-01-26

**Authors:** Mira Okshevsky, Viduthalai R. Regina, Ian P. G. Marshall, Lars Schreiber, Rikke L. Meyer

**Affiliations:** a Interdisciplinary Nanoscience Center (iNANO), Aarhus University, Aarhus, Denmark; b Center for Geomicrobiology, Section for Microbiology, Department of Bioscience, Aarhus University, Aarhus, Denmark; c Microbiology Section, Department of Bioscience, Aarhus University, Aarhus, Denmark

## Abstract

Representatives of the genus *Bacillus* are common milk contaminants that cause spoilage and flavor alterations of dairy products. *Bacillus* sp. FMQ74 was isolated from raw milk on a Danish dairy farm. To elucidate the genomic basis of this strain’s survival in the dairy industry, a high-quality draft genome was produced.

## GENOME ANNOUNCEMENT

*Bacillus* spp. are Gram-positive bacteria commonly associated with spoilage and flavor alterations of finished dairy products. They are found in raw milk and subsequent stages of milk processing (1, 2) and can cause food poisoning in humans (3, 4). The ability of *Bacillus* spp. to form communities called biofilms greatly contributes to their success as dairy contaminants. Biofilms are difficult to eradicate from food-processing surfaces due to their recalcitrance to antimicrobials and common cleaning procedures (5, 6). A survey of milk contaminants on 49 dairy farms in Denmark revealed that the FMQ74 operational taxonomic unit (OTU) was present in raw milk on 29 farms. *Bacillus* sp. FMQ74 was therefore selected for sequencing due to its ubiquitous presence as a milk contaminant.

*Bacillus* strain FMQ74 was isolated from raw milk. The milk was heated to 63°C for 30 min, diluted 1:10 with tryptic yeast extract broth, and incubated for 24 h at 30°C. The enriched milk was serially diluted, inoculated onto tryptic soy broth agar plates, and incubated at 30°C. The single colony was restreaked six times prior to genomic DNA extraction using the GeneJet genomic DNA extraction kit (Thermo Scientific). A sequencing library was prepared using the Nextera XT kit (Illumina, USA). Genome sequencing was performed using the Illumina MiSeq platform with a paired-end 300-bp MiSeq reagent kit version 3, resulting in ca. 4.4 million sequencing reads representing 1.2 Gbp and an approximately 250× coverage. Reads were quality inspected using FastQC version 0.11.5 (http://www.bioinformatics.babraham.ac.uk/projects/fastqc/) and were quality and adapter trimmed using Trimmomatic version 0.36 (7). Quality-based trimming used a 4-bp sliding window and minimum average quality score ≥20. Reads >200 bp were assembled using SPAdes 3.9.0 (8) with the parameters: --careful –k 21, 33, 55, 77, 99, 127, resulting in 65 contigs (4,167,316 bp). Only contigs with G+C content between 20% and 65% and coverage between 10× and 3,500× (determined using BBmap version 35.82 [https://sourceforge.net/projects/bbmap/]) were retained, resulting in 52 contigs representing 99.9% of the original assembly. Contigs identified as contamination by 16S comparison with the SILVAngs database release 128 (9) were removed, leaving 47 contigs in the final draft genome assembly. This assembly was manually augmented with 849 bp of the 16S gene PCR amplified from genomic DNA and Sanger sequenced.

The draft genome sequence *Bacillus* sp. FMQ74 has a total length of 4,159,532 bp, an average G+C content of 43.3%, and an *N*_50_ length of 345,666 bp. The genome was estimated to be 99.4% complete compared to the single-gene marker set for the genus *Bacillus* via CheckM 1.0.7 (10). Prokka 1.12-beta (11) identified 4,204 protein-coding sequences, 13 rRNA sequences, and 84 tRNA sequences. This genome contains the complete *epsA-O* operon encoding the major polysaccharide of the *Bacillus subtilis* biofilm extracellular matrix (12), the *tapA-sipW-tapA* operon encoding amyloid protein (13–15), biofilm regulatory proteins *sinI* and *sinR* (16), as well as the poly-γ-dl-glutamic acid biosynthesis genes required for submerged biofilm formation in *B. subtilis* (17).

### Accession number(s).

This draft genome sequence has been deposited at DDBJ/ENA/GenBank under the accession number MOEO00000000. The version described here is version MOEO01000000.
